# Hole-Transport Material Engineering in Highly Durable Carbon-Based Perovskite Photovoltaic Devices

**DOI:** 10.3390/nano13081417

**Published:** 2023-04-20

**Authors:** Reza Rahighi, Somayeh Gholipour, Mohammed A. Amin, Mohd Zahid Ansari

**Affiliations:** 1 SKKU Advanced Institute of Nano-Technology (SAINT), Sungkyunkwan University, 2066, Seobu-ro, Jangan-gu, Suwon 16419, Republic of Korea; 2 Adolphe Merkle Institute, Chemin des Verdiers 4, CH-1700 Fribourg, Switzerland; 3 Department of Chemistry, College of Science, Taif University, P.O. Box 11099, Taif 21944, Saudi Arabia; 4 School of Materials Science and Engineering, Yeungnam University, Gyeongsan 712749, Republic of Korea

**Keywords:** composition engineering, carbon fibers, durability, cost-efficiency, lifetime

## Abstract

Despite the fast-developing momentum of perovskite solar cells (PSCs) toward flexible roll-to-roll solar energy harvesting panels, their long-term stability remains to be the challenging obstacle in terms of moisture, light sensitivity, and thermal stress. Compositional engineering including less usage of volatile methylammonium bromide (MABr) and incorporating more formamidinium iodide (FAI) promises more phase stability. In this work, an embedded carbon cloth in carbon paste is utilized as the back contact in PSCs (having optimized perovskite composition), resulting in a high power conversion efficiency (PCE) of 15.4%, and the as-fabricated devices retain 60% of the initial PCE after more than 180 h (at the experiment temperature of 85 °C and under 40% relative humidity). These results are from devices without any encapsulation or light soaking pre-treatments, whereas Au-based PSCs retain 45% of the initial PCE at the same conditions with rapid degradation. In addition, the long-term device stability results reveal that poly[bis(4–phenyl) (2,4,6–trimethylphenyl) amine] (PTAA) is a more stable polymeric hole-transport material (HTM) at the 85 °C thermal stress than the copper thiocyanate (CuSCN) inorganic HTM for carbon-based devices. These results pave the way toward modifying additive-free and polymeric HTM for scalable carbon-based PSCs.

## 1. Introduction

Solar energy conversion is growing dramatically as the feasible solution to meet the global demand of securing sustainable energy and new strategies for inexpensive and clean energy alternatives. Having reached high power conversion efficiencies (PCEs) up to a certified value of ~32.5% (in silicon-tandem configuration structures) [1], perovskite solar cells (PSCs) have proved to be a reasonable solution regarding most of the energy concerns of the future and make their way toward industrial production in the current decade [2]. The general chemical form of perovskites is ABX_3_, where the A-site ion occupies a central cavity enclosed by corner-shared octahedral BX_6_ units forming a cubic crystal structure, where A = cesium (Cs), methylammonium (MA), or formamidinium (FA); B = tin (Sn) or lead (Pb); X = chlorine (Cl), bromine (Br), or iodine (I). By interchanging the as-mentioned cations [3,4], metals [5,6], or halides [7], bandgap can be tuned in a favorable range of 1.2 to 3 eV. Being sensitive to oxidative degradation, the long-term stability on the module level under real outdoor working conditions is one of the main issues hampering the commercialization of PSCs [8,9,10].

Material engineering of PSCs has resulted in their PCE increase from 3.8% to 25.7% in about 10 years from their first practical module [1,11] thanks to the outstanding photoelectric properties of perovskite single crystals, such as large absorption coefficients (over 10^5^ cm^−1^ for MApbI_3_ having a bandgap of 1.57 eV, with an Urbach energy as small as 15 meV) [12], ambipolar charge transport [13] with negligible exciton binding energy [14], long-charge carrier diffusion lengths [15], and high defect tolerance [16]. To achieve PCE > 22%, most devices use costly organic small-molecule hole-transport materials (HTMs) with dopants and noble metals such as gold or silver for the back electrodes, which has drawbacks of instability due to crystallization and gold migration at temperatures higher than 70 °C [17].

The unwanted structural phase transition from tetragonal to cubic of single-cation perovskite at the relatively low temperature of 55 °C and abrupt change of the ionic/electrical rate due to a change in the ion activation energy from 0.7 to 0.5 eV at 45 °C [18] also causes serious concern regarding their applications [19]. Moreover, nanoscale phase impurities activate degradation sites in halide perovskites, which requires careful tuning of local structural and chemical properties of perovskite materials [20,21]. Additionally, as reported previously, a lower amount of volatile MA and more FA inhibit the thermal instability and lower Br/I ratios can stop bandgap blue shifting by tuning it to benefit higher current densities [22]. Thus, compositional engineering facilitates overcoming perovskite phase instability [23].

The high thermal conductivity of carbon-based electrodes has been reported to be an alternative owing to material abundance [24], low cost [25], industrially mature preparation [26], biocompatibility [27], and excellent stability against humidity and temperature [28] (within the solar cell range of working conditions). There have been various reports on carbon-based PSCs, where high PCEs were obtained by using spiro-OMeTAD [2,2′,7,7′-tetrakis(*N*,*N*-di-p-methoxyphenyl-amine)9,9′-spirobifluorene] [29], or a polymer-based poly(triarylamine) (PTAA) [30], or a copper thiocyanate (CuSCN) [31] as the hole-transporting material (HTM). Despite the demonstrated photovoltaic (PV) performances, the controllability of the doping process and shelf-life stability (lifetime) of doped HTMs remain largely untapped. The dopant-free HTMs such as CuSCN can be utilized as an alternative HTM, in contrast to PTAA and spiro-OMeTAD, which need a p-dopant [32] such as lithium salt and 4-*tert*-butylpyridine and, for the latter, also a Co(III) complex to reach their best performance. It is worth mentioning that PTAA used a lower concentration of dopants compared to Spiro-OMeTAD, and thus it can result in more stable devices. There were calls for the coordination of Li salts with alkylthiol (1-dodecanethiol, DDT) to inhibit the dopant accumulation at interfaces, where the structural integrity of the HTM under moisture, heat, and light stress was improved [33]. Moreover, Chu et al. reported carbon-based devices with Poly(3-hexylthiophene-2,5-diyl) (P3HT)/graphene composite HTM [34], which resulted in a stabilized efficiency of 17.8% and encapsulated devices retain 89% of their original performance after 600 h (under 1-sun illumination). However, the pristine P3HT (graphene-free) HTM-based devices showed a much lower PCE of 11% undergoing rapid degradation, reaching ~25% of its original output after ~75 h under the same condition. Although the incorporation of the costly graphene pathways into the P3HT HTM was introduced as a strategy to increase hole mobility, the thermal tolerance of the devices at 85 °C standard testing temperature was not investigated [34].

Metallic back contacts, such as physical-vapor-deposited silver or gold react with iodine-containing perovskite compounds to form (AuI_2_)^−^ and AuI_3_, resulting in faster degradation of the gold electrode. Moreover, UV-light-induced decomposition of perovskite e.g., MAPbI_3_ leads to a rapid reaction with gold resulting MA_2_Au_2_I_6_ phase with a tetragonal symmetry, which can penetrate through hole transport layers (HTLs), and participate in the degradation of the devices [17,35,36,37]. Therefore, gold is considered an unsuitable back contact for wide use in iodine-based PSCs; to overcome this issue, cost-efficient carbon-based back electrodes are attracting attention thanks to their effectiveness in boosting the stability of PSCs.

Herein, the perovskite composition is tuned in terms of a higher ratio of FAI to MABr to use less volatile MA and gain more current density as a result of less Br to I, thus adjusting to the smaller bandgap. Moreover, the stability of the modified carbon-based PSCs based on Spiro-OMeTAD, PTAA, and CuSCN HTM is investigated. Additionally, the nature of degradation of the carbon-based and control gold-based devices is also investigated under 40% relative humidity (RH) at 85 °C and in the dark, where the large molecules HTMs (PTAA) retained higher initial PCE under the shelf life stability testing condition.

## 2. Materials and Methods

Anhydrous ethanol, isopropyl alcohol (IPA), acetylacetonate, hydrochloric acid (HCl), Hellmanex aqueous solution, bis(trifluoromethylsulfonyl)imide lithium salt (LiTFSI), dimethylformamide (DMF), dimethyl sulfoxide (DMSO), diethyle sulfide (98%), and chlorobenzene were purchased from Sigma-Aldrich, as was fluorine-doped tin-oxide (FTO)-coated glass (TEC-7 Ω sq^−1^), poly(methyl methacrylate (PMMA), and 4-tert-butylpyridine (TBP). TiO_2_ paste (with 30 nm particle size) and organic cation iodide salts were purchased from GreatCell Solar (30 NR-D). Lead compounds, cesium iodide (ultra-dry CsI, 99.998%), and spiro-OMeTAD were bought from TCI, GmbH, and Luminescence Technology, respectively. The commercial conductive carbon paste was purchased from Solaronix (Elcocarb B/SP).

### 2.1. Preparation of TiO_2_ Compact and Mesoporous Layers

The FTO-coated glass was firstly etched chemically via zinc powder and 3 M HCl and cleaned with Hellmanex aqueous solution (2%), ethanol, and IPA via separate consequent sonication process (30 min), followed by a 10 min UV-ozone treatment. Spray pyrolyzed TiO_2_ compact layers were deposited at 450 °C (30 nm) from a precursor solution of titanium di-isopropoxide bis(acetylacetonate) in anhydrous ethanol and acetylacetonate, followed by annealing the substrates at (30 min at 450 °C). Then, diluted mesoporous TiO_2_ paste (100 mg mL^−1^ in ethanol) was spin-coated. To achieve a thickness of ~ 150 nm, the rpm, ramp, and time were set to be 4000 and 2000 rpm s^−1^ and 10 s, respectively. The substrates were immediately dried at 100 °C for 10 min and then sintered at 450 °C (30 min under a dry airflow). It should be noted that 450 °C is known as a suitable temperature for anatase phase formation of the TiO_2_ layer working as an electron transport layer [38]. Then, LiTFSI solution in acetonitrile with a density of 10 mg mL^−1^ was spin-coated (3000 rpm, 1000 rpm s^−1^, 10 s) on the mesoporous scaffold to increase the ETL conductivity, followed by a second sintering step (with same conditions). After cooling down to 150 °C, the substrates were transferred into the nitrogen-filled glove box to inhibit moisture ingress to mesoporous TiO_2_ layers.

### 2.2. Perovskite Precursor Solution and Deposition

PbI_2_ (1.1 M) in anhydrous DMF: DMSO, 4:1 (*v*:*v*) was poured onto FAI (1 M) to result in FAPbI_3_ solution. A precursor solution of PbBr_2_ (0.2 M) in anhydrous DMF: DMSO, 4:1 (*v*:*v*) was added to MABr (0.2 M) to form the MAPbBr_3_. The FAPbI_3_ and MAPbBr_3_ solution was mixed with CsI solution (1.5 M solution of CsI powder in DMSO) in an adjusted stoichiometry to form a chemical composition of Cs_0.5_(MA_0.17_FA_0.83_)_0.95_Pb(I_0.83_Br_0.17_)_3_ or Cs_0.5_(MA_0.10_FA_0.90_)_0.95_Pb(I_0.90_Br_0.10_)_3_ [39]. The perovskite solution was spin-coated on a mesoporous TiO_2_ layer by an antisolvent process at 3000 r.p.m for 30 s and ramp of 2000 r.p.m. s^−1^, where 100 μL of chlorobenzene was dropped slowly on the substrate during the last 10 s of the program. To complete the perovskite layer crystallization process, the substrates were then heated at 100 °C for 45 min in a nitrogen-filled glove box.

### 2.3. PMMA Precursor Solution

To passivate the perovskite grain boundaries and perovskite/HTM interface, a 0.1 mg PMMA (MW: 120,000) in 1 mL chlorobenzene was prepared. Following the perovskite annealing, the substrates were cooled down to room temperature, and the PMMA solution was spin-coated dynamically at 4000 r.p.m., for 30 s, on the perovskite layer.

### 2.4. Hole-Transporting Layers

The spiro-OMeTAD solution was spin-coated dynamically on a rotating substrate at 4000 rpm for 20 s. In summary, the 70 mM Spiro-OMeTAD precursor solution was prepared by dissolving 92 mg Spiro-OMeTAD in 1 mL chlorobenzene with the additives 21 μL LiTFSI stock solution (520 mg Li-TFSI, in 1 mL Acetonitrile), 28.6 μL TBP, and 15 μL stock solution cobalt (III) tris(bis(trifluoromethylsulfonyl)imide) (31 mg FK209, Dyesol, in 84 μL Acetonitrile), as reported elsewhere [40]. The PTAA precursor comprising 10 mg PTAA powder, was completely dissolved in 1 mL toluene, followed by adding 16 μL Li-TFSI in acetonitrile (180 mg mL^−1^) and 20 μL TBP (140 mg in 1 mL toluene) to main solution, which was then spin-coated at 3000 rpm for 30 s. The 3.5 mg CuSCN precursor was added to 1 mL diethyle sulfide, followed by 30 min of stirring to solubilize all the precursors, which was then spin-coated dynamically on the spinning substrate at 5000 rpm for 30 s.

### 2.5. Back Contact

**Figure 1:**
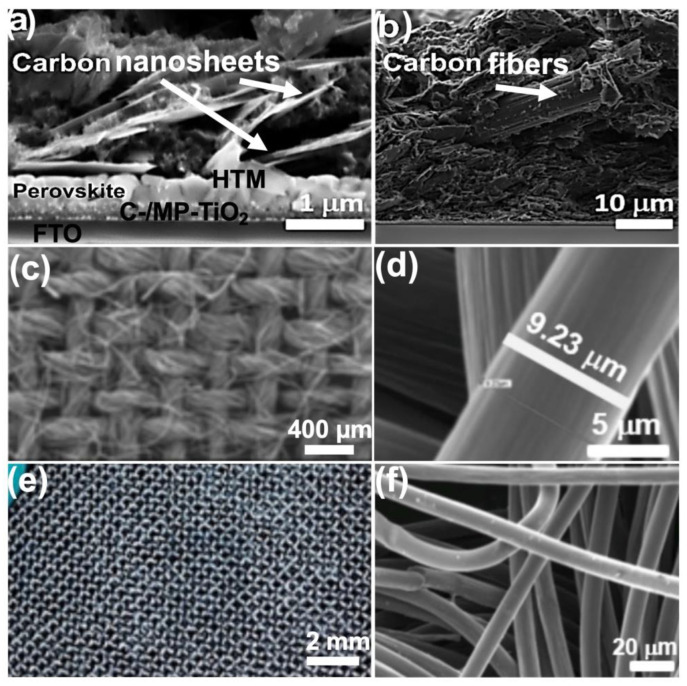
Cross-sectional FE-SEM images of the carbon-based PSCs (**a**), CC embedded in carbon paste/spiro–OMeTAD composition (**b**). Overview FE-SEM image of CC (**c**), and close-up image of a single fiber of the used CC (**d**). Digital image of CC (**e**); typical diameter of each carbon fiber is 9 μm according to the FE-SEM image of CC texture (**f**).

For Au-based (control) devices, 80 nm of the gold electrode was thermally evaporated by the physical vapor deposition (PVD) method under 10^−7^ mbar vacuum pressure. The active area of the metal mask was 0.16 cm^2^. For carbon-based devices, as described elsewhere [41], an optimized thickness of ≈20 μm carbon layer was deposited as a back electrode. In summary, carbon paste was dried (130 °C, 2 h) for solvent exchange to chlorobenzene (0.4 g ml^−1^) to become compatible with the perovskite layer. The carbon cloth (CeTech Co, without MPL&PTFE, Figure 1c–f) was pressed (by a binder clip) on the doctor-bladed carbon paste. After drying the whole cells at 70 °C for 10 min, the binder clips and thick CC (0.3 mm) were removed, where only a thin layer of 10 μm carbon fibers was incorporated in the carbon paste (verified by FE-SEM cross-section images). The densification of back contact was conducted by drop casting 3 μL of the doped Spiro—OMeTAD, doped PTAA, or CuSCN HTM, on each pixel, followed by 70 °C heating for 2 min. Subsequently, silver paint (Leitsilber 200N) was added to the edge of the carbon back contact.

### 2.6. Device Characterization

All PV devices were analyzed with a solar simulator from ABET Technologies (Model Sun 2000) with a xenon arc lamp (100 mW cm^−2^, AM 1.5 G), and the solar cell response was recorded using a Metrohm PGSTAT302N Autolab. The light intensity was calibrated using a silicon reference cell from ReRa Solutions (KG5 filtered). Current–voltage curves were measured in a scan rate of 20 mV s^−1^ at the reverse bias. To adjust the active optical area and reduce the influence of the scattered light, the cells were masked with a black metal mask with an 0.09936 cm^2^ aperture. Field emission scanning electron microscopy (FE-SEM) was performed with a Tescan MIRA 3 LMH at an acceleration voltage of 10 kV. For stability measurements, the unencapsulated gold and carbon-based devices were aged under 85 °C and ambient humidity (relative humidity of approximately 40%) without light soaking.

## 3. Results and Discussion

A variety of high-conductive CC with great resistance to fatigue, heat, and corrosion are facilely manipulated as a back contact for different electronic devices. Herein, a modified CC was utilized as the carbon back contact. In this regard, a piece of CC was used to effectively increase the conductivity of back contact. The cross-sectional FE-SEM images of the carbon-based solar cell are shown in Figure 1a. In all different types of prepared cells, the thicknesses of the perovskite/infiltrated mesoporous TiO_2_ layer and the perovskite capping layer are almost 150 nm and 377 nm, respectively. The morphology of the carbon layers, including carbon sheets and embedded carbon fibers in carbon paste, is shown in Figure 1b. Accordingly, the thickness of the carbon paste layer as well as the CC embedded in the carbon paste is around 20 μm. Figure 1c,d demonstrate specific features of CC mesh and its fine fibers having a diameter of ~10 μm, and more details from its cross-section FE-SEM investigations are given in Figure 1f. An overview digital image of the utilized CC is provided in Figure 1e as well.

**Figure 2:**
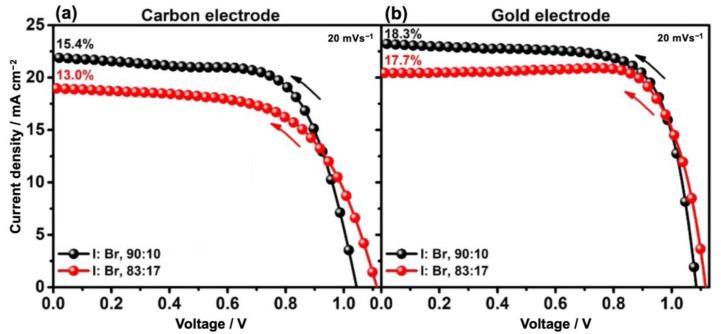
J-V curves of the champion perovskite devices with different back contacts. The CC embedded in carbon paste/spiro–OMeTAD cell (**a**) and spiro–OMeTAD/Au device (**b**). The backward J-V scan direction (at 20 mV s^−1^ scan rate) is shown by arrows.

In this work, by tuning the amount of MABr over FAI, more efficient carbon-based solar cells were fabricated. As shown in Figure 2a and Table 1, the record backward PCE of 15.4% was obtained for the tuned amount of I: Br, which was higher than that of the higher amount of the MABr (PCE of 13%) for carbon-based devices. Hence, the 90:10 ratio was adjusted for the case of I: Br composition as a reference in optimizing the carbon-based devices.

**Figure 3:**
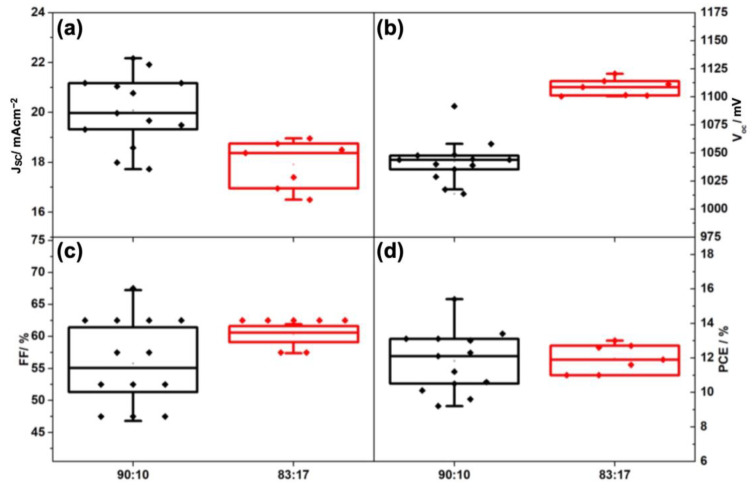
Statistics of PV parameters, namely J_SC_ (**a**), V_OC_ (**b**), FF (**c**), and PCE (**d**), of carbon-based solar cells with two different ratios of I: Br, 90:10 and 83:17.

Figure 3 and Table 2 show the statistical PV parameters of carbon-based solar cells with different ratios of FAI: MABr. As shown in Figure 3, using a lower amount of MABr (10%) resulted in a higher average short circuit current density (J_sc_) (20.1 mA cm^−2^) because of the lower bandgap of 1.6 eV [42] compared to a higher amount of MABr (17%) with E_g_ of 1.63 eV [22] and higher absorption of incident photons in the visible region, and accordingly, a lower average open circuit voltage (V_oc_) (1042.5 mV) was obtained (Table 2). Therefore, the average PCE of the ratio of 90:10, I: Br ratio was on par with the ratio of 83:17 obtained at ~12%.

**Figure 4:**
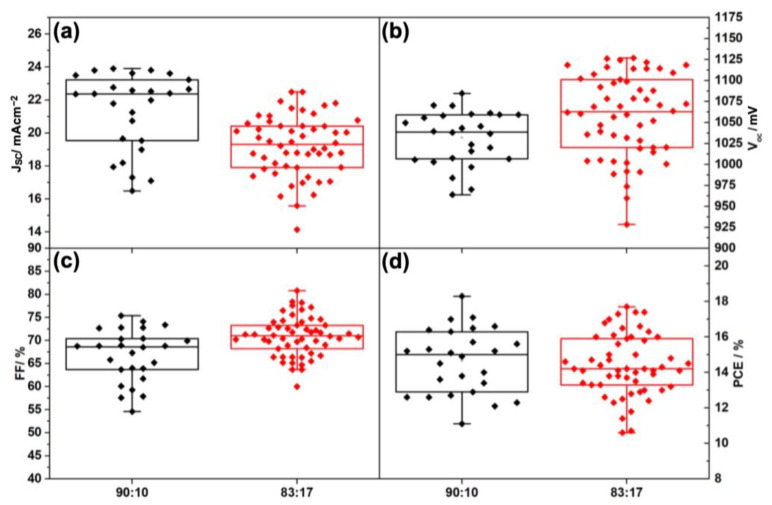
Statistics of PV parameters: J_SC_ (**a**), V_OC_ (**b**), FF (**c**), and PCE (**d**) of gold-based solar cells with different ratios of I: Br of 90:10 and 83:17.

The same features have been observed and discussed in Figure 4 and Table 2 for control devices with gold back contact. Using a lower amount of MABr resulted in a higher average J_sc_ (21.3 mAcm^−2^) and lower average V_oc_ (1031.86 mV) and the average PCE remained almost the same (~14.5%). As shown in Figure 2b and Table 1, the record PCE for gold-based devices was higher for the tuned amount of MABr (18.3 to 17.7%).

Although the higher content of FAI over MABr has the advantage of gaining more short circuit current density (J_SC_) and more stable devices, further increase > 90:10 of FAI: MABr was not investigated here due to the following two drawbacks: (i) pure FAPbI_3_ lacks structural stability at room temperature results in the formation of the non-perovskite hexagonal δ-phase (yellow phase), in contrast to photoactive perovskite α-phase (black phase) [43], which is sensitive to solvents or humidity [44]; and (ii) lower bromide redshifts the perovskite bandgap, resulting in the lower V_OC_. Therefore, mixing the cation (or halide) dramatically improves the perovskite film morphology and optoelectronic properties.

**Figure 5:**
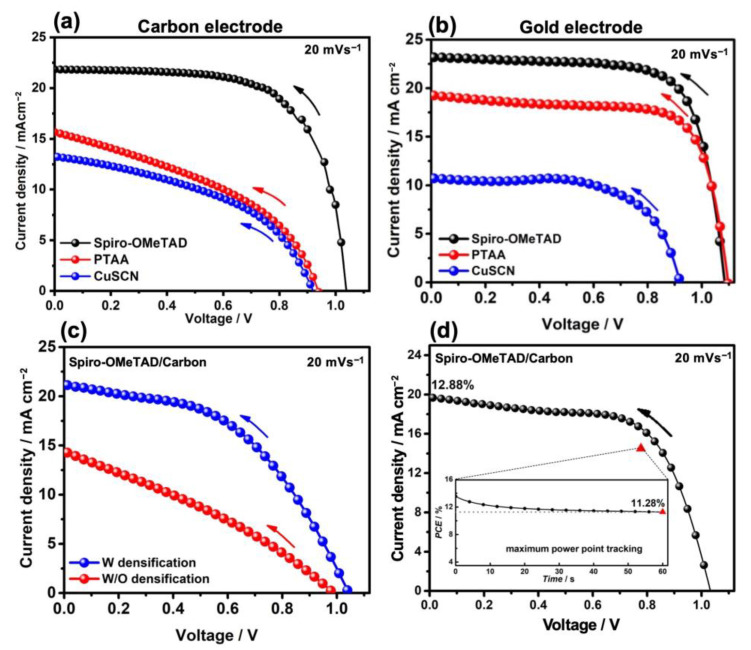
The J-V curves of the carbon-based solar cell types (**a**). Au-based solar cells fabricated by different HTLs (**b**). Spiro OMeTAD/carbon or gold cell in black, PTAA/carbon or gold in red, and CuSCN/carbon or gold device in blue. The arrows indicate the backward J-V scan direction at 20 mV s^−1^. The J-V curves of the different carbon-based solar cell types (**c**); Spiro-OMeTAD/carbon composition/Spiro-OMeTAD cell (with densification) in blue and Spiro-OMeTAD/carbon composition device (without densification) in red. Backward J-V curve and MPP tracking of the carbon-based solar cells (**d**). The voltage scan rate for all of the scans was 20 mV s^−1^.

**Figure 6:**
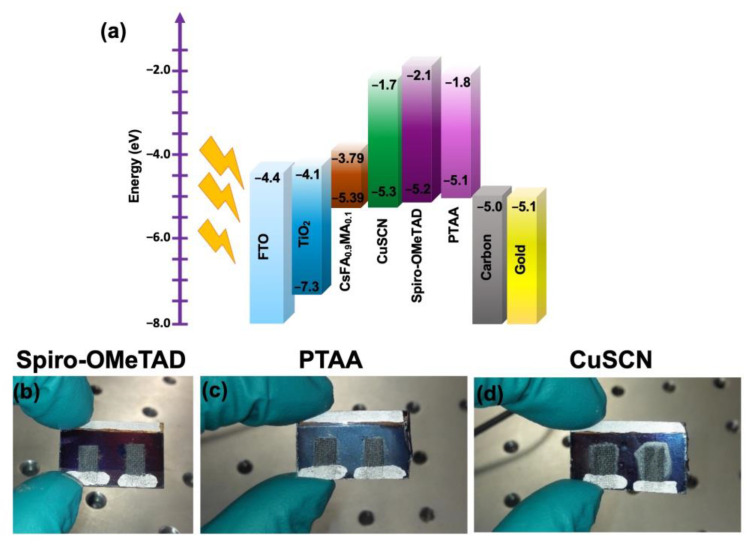
The corresponding energy band diagram for the carbon, or gold-based devices with three different HTMs (**a**). The optical images of the Spiro-OMeTAD/carbon (**b**), PTAA/carbon (**c**), and CuSCN/carbon-based devices (**d**).

**Figure 7:**
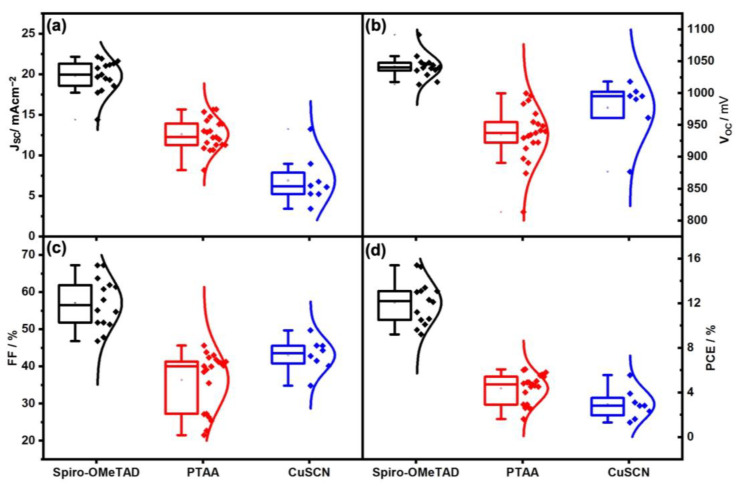
Statistics of J_SC_ (**a**), V_OC_ (**b**), FF (**c**), and PCE (**d**) related to carbon-based solar cells with different HTMs and densification layers, including Spiro-OMeTAD, PTAA, and CuSCN.

**Figure 8:**
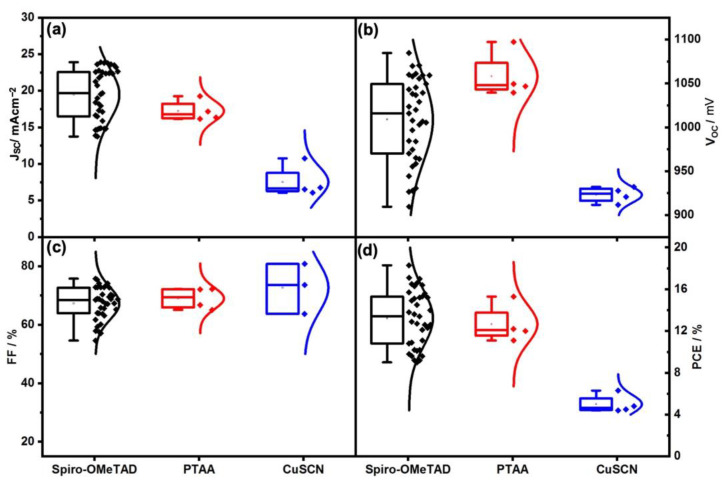
Statistics of J_SC_ (**a**), V_OC_ (**b**), FF (**c**), and PCE (**d**) related to gold-based solar cells with different HTMs and densification layers, including Spiro-OMeTAD, PTAA, and CuSCN.

In addition, the effect of different HTMs was investigated including Spiro-OMeTAD, PTAA, and CuSCN on the carbon- (see Figure 5a and Figure 6b–d) and gold-based PSCs (Figure 5b). As shown in record performance (see Figure 5a, and Table 3) and the statistical PV parameters (see Figure 7 and Table 4), using Spiro-OMeTAD resulted in higher PV parameters rather than the PTAA and CuSCN. Moreover, using gold-based devices, different HTMs were tried including Spiro-OMeTAD, PTAA, and CuSCN (data presented in the record J-V curves in Figure 5b and Table 3, and statistical PV parameters are presented in Figure 8 and Table 4). The gold-based devices showed the same trend regarding the PV parameters as the carbon counterparts. As can be seen in the energy band diagram (Figure 6a) of the carbon and gold-based devices, PTAA and spiro-OMeTAD have more favorable band alignment with back contact rather than CuSCN HTM, [42,45] which results in higher PV performance.

The proper densification of the carbon fibers into the carbon paste can be obtained by dropping a small amount (3 μL) of HTM on top of the back contact. The densification process increased all PV parameters in carbon-based devices, as shown in Figure 5c and Table 5. The record PCE for the carbon-based devices increased from 4.4 to 10.6% by applying a densification HTM (Spiro-OMeTAD) to the back contact. Moreover, the carbon-based devices showed a low hysteresis, as can be seen from Figure 5d and Table 6, and the maximum power point tracking (MPP) PCE value (11.28%) was on par with the one obtained from backward J-V scan direction (12.88%).

**Figure 9:**
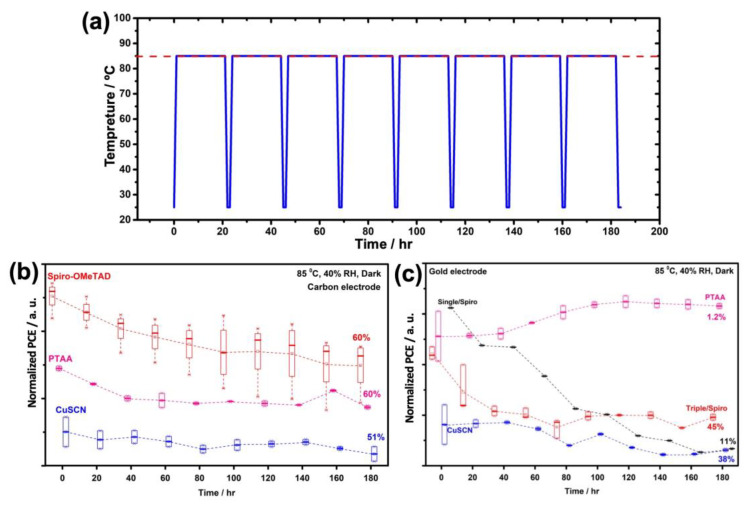
Profile of thermal cycling in stability tests (**a**)**.** Evaluation of device performance from the stability test of PSCs based on carbon back contact without encapsulation under 85 °C and 40% RH without light soaking (**b**). Evaluation of device performance from the stability test of PSCs based on gold back contact with different HTLs, including PTAA, Spiro-OMeTAD, CuSCN, and without encapsulation under 85 °C and 40% RH without light soaking (**c**); (triple states for triple cation perovskites and single states for single cation perovskites and MAPbI_3_).

The promising advantage of the application of carbon as the back electrode instead of its metallic counterparts are demonstrated to enhance the stability of solar cells [46,47]. The long-term device stability of the carbon-based PSCs with different HTMs was further investigated under the thermal cycling profile for the stability test as shown in Figure 9a. The temperature of devices was kept at 85 °C, and the PV measurements were performed at room temperature (~25 °C). As can be seen in Figure 9b, carbon-based devices which were fabricated by Spiro-OMeTAD and CuSCN HTMs retained 60 and 51% of their initial efficiencies, respectively under an ambient environment with an average 40% RH, 85 °C temperature, and dark condition after 180 h.

However, the gold counterparts underwent severe degradation under the same condition and retained 45 and 38% of their initial efficiencies, respectively (see Figure 9c). In the case of using PTAA as HTM, the carbon-based devices retained 60% of their initial efficiency, the same as the spiro-OMeTAD counterparts, which mainly arise from the lower hole extraction of devices, which accelerates the electron-hole recombination at the PTAA/carbon interfaces. In contrast, the PCE of gold-based devices fabricated by PTAA HTM underwent an increase of almost 1.2% after 180 h under 40% RH and 85 °C temperature (see Figure 9c), which is related to two mechanisms: (i) PTAA as an HTM provides excellent interface passivation for the buried surface trap states and defects of perovskite layer [30,48,49,50] and (ii) hydrophobic nature PTAA protects the perovskite from moisture, thus improving the stability [51,52].

In fact, a potentially cost-effective CC embedded into the carbon paste composition deposited directly onto the perovskite/PMMA/HTM layer (and processed further with a small amount of HTM) yielded a PCE of 15.3, 6.1, and 5.3% for Spiro-OMeTAD, PTAA, and CuSCN HTMs. The stability of such carbon-based devices under 85 °C, 40% RH, and dark conditions was also proved to be improved compared with typical gold counterparts under similar stability tests. Moreover, as expected from the importance of compositional engineering, the gold-based PSCs fabricated by using single cation perovskites (MAPbI_3_) degraded much faster than the triple cation counterparts and retained only 11% of their initial PCE under the same condition (see Figure 9c).

It is worth mentioning, there are several highly conductive commercial carbon pastes for screen printing and blade coating, which are composed of different sizes of carbon black, carbon flakes, curing resin or organic binders, and organic solvents. The composition of the carbon paste plays a crucial role in the device performance and stability of the carbon-based PSCs, where the best-performing carbon pastes have faster processing at lower temperatures due to faster solvent evaporation and binder removal. Although the solvents we used here for the carbon paste (chlorobenzene) have been proven to be compatible with the perovskite in our previous work, [40] it may still have some influence on the quality of the spiro-OMeTAD HTL and related interfaces. Therefore, we believe that using low-temperature-carbon pastes with harmless solvents preserves dense and uniform morphology, resulting in lower interfacial resistance and higher performance and stability.

## 4. Conclusions

In summary, the instability challenges caused by external environmental factors including thermal stress and humidity were addressed by tuning-temperature-resilient FAI over MABr in perovskite composition and humidity-resistant carbon fiber embedded in the carbon paste back contact. The prepared devices with a record PCE of 15.4% showed only a modest linear efficiency loss (at 85 °C, in an ambient atmosphere of 40% RH, and a dark condition of the experiment), which led to an estimated lifetime (T80) of 180 h, whereas the control PSCs with gold back contact experienced a dramatic and rapid efficiency loss. The main reason for the fast degradation of gold-based devices was related to the Au ion migration through HTM and rapid reaction with the iodine-based perovskite structure. Using a polymeric hole extraction layer such as PTAA in the design of PSCs can result in high PCE values thanks to the trap state passivation effect and hydrophobic nature that assisted the enhanced thermal stability.

## Figures and Tables

**Table 1:** The PV parameters of the champion devices with different back contacts (Au and carbon) and halide ratios. PCE_(max)_ stems for the record efficiencies scanned in the backward direction and 20 mV s^−1^ scan rate.

I:Br	Back Contact	V_OC_ (V)	J_SC_ (mAcm^−2^)	FF (%)	PCE _(max)_ (%)
90:10	Gold	1.09	23.23	72.8	18.34
	Carbon	1.04	21.92	67.2	15.37
83:17	Gold	1.12	20.42	77.6	17.69
	Carbon	1.11	18.96	61.6	13.02

**Table 2:** The average PV parameters and standard deviations of the carbon- and gold-based devices with different halide ratios.

I:Br	Back Contact	V_OC (avg)_ (mV)	J_SC (avg)_ (mAcm^−2^)	FF _(avg)_ (%)	PCE _(avg)_ (%)
90:10	Gold	1031.86 ± 32.29	21.31 ± 2.36	66.79 ± 5.66	14.64 ± 1.84
	Carbon	1042.47 ± 19.24	20.08 ± 1.44	55.79 ± 6.78	11.82 ± 1.79
83:17	Gold	1058.05 ± 48.90	19.21 ± 1.82	70.86 ± 4.26	14.39 ± 1.71
	Carbon	1108.15 ± 7.65	17.92 ± 0.96	60.34 ± 1.61	11.97 ± 0.82

**Table 3:** J-V parameters of the champion Au and carbon-based devices with different HTMs, including Spiro-OMeTAD, PTAA, and CuSCN.

HTM	Back Contact	V_OC_ (V)	J_SC_ (mA cm^−2^)	FF (%)	PCE _(max)_ (%)
Spiro-OMeTAD	Gold	1.09	23.23	72.8	18.3
	Carbon	1.04	21.83	67.3	15.3
PTAA	Gold	1.01	19.27	72.2	15.3
	Carbon	0.94	15.66	41.2	6.1
CuSCN	Gold	0.92	10.75	63.7	6.3
	Carbon	0.92	13.25	45.5	5.6

**Table 4:** The average and standard deviations of PV parameters of the carbon-based devices with different HTMs and the densification material, including Spiro-OMeTAD, PTAA, and CuSCN.

HTM	Back Contact	V_OC (avg)_ (mV)	J_SC (avg)_ (mA cm^−2^)	FF _(avg)_ (%)	PCE _(avg)_ (%)
Spiro-OMeTAD	Gold	1009.03 ± 47.98	19.49 ± 3.52	67.29 ± 5.57	13.23 ± 2.71
	Carbon	1041.76 ± 18.56	19.81 ± 2.05	57.10 ± 6.77	12.06 ± 1.96
PTAA	Gold	1058.29 ± 26.31	17.24 ± 1.42	69.03 ± 3.66	12.65 ± 1.83
	Carbon	935.12 ± 43.52	12.60 ± 1.93	36.34 ± 7.70	4.35 ± 1.32
CuSCN	Gold	923.10 ± 8.94	7.52 ± 2.17	72.7 ± 8.59	5.00 ± 0.88
	Carbon	967.67 ± 51.29	6.90 ± 3.01	43.03 ± 4.43	2.92 ± 1.35

**Table 5:** J-V parameters of the carbon-based devices with and without densification by spiro-OMeTAD. PCE_(max)_ stems for the record efficiency scanned in the backward direction and 20 mV s^−1^ scan rate.

Densification by HTM	V_OC_ (V)	J_SC_ (mAcm^−2^)	FF (%)	PCE_(max)_ (%)
Without	0.99	14.39	31.3	4.4
With	1.04	21.17	47.8	10.6

**Table 6:** J-V parameters of the carbon-based devices fabricated by spiro-OMeTAD. The record efficiency in the backward scan direction is marked with PCE_max_.

I:Br	V_OC_ (V)	J_SC_ (mAcm^−2^)	FF (%)	PCE_(max)_ (%)
90:10	1.03	19.70	63.32	12.88

## Data Availability

The data presented in this study are available on request from the corresponding author.
